# The Actuation Mechanism of 3D Printed Flexure-Based Robotic Microtweezers

**DOI:** 10.3390/mi10070470

**Published:** 2019-07-14

**Authors:** Alexander Almeida, George Andrews, Devina Jaiswal, Kazunori Hoshino

**Affiliations:** 1 Department of Biomedical Engineering, University of Connecticut, Storrs, CT 06269, USA; 2 Department of Biomedical Engineering, Western New England University, Springfield, MA 01119, USA

**Keywords:** manipulator, flexure, 3D-printing, micro/meso-scale manipulation, piezo bimorph actuator

## Abstract

We report on the design and the modeling of a three-dimensional (3D) printed flexure-based actuation mechanism for robotic microtweezers, the main body of which is a single piece of nylon. Our design aims to fill a void in sample manipulation between two classes of widely used instruments: nano-scale and macro-scale robotic manipulators. The key component is a uniquely designed cam flexure system, which linearly translates the bending of a piezoelectric bimorph actuator into angular displacement. The 3D printing made it possible to realize the fabrication of the cam with a specifically calculated curve, which would otherwise be costly using conventional milling techniques. We first characterized 3D printed nylon by studying sets of simple cantilevers, which provided fundamental characteristics that could be used for further designs. The finite element method analysis based on the obtained material data matched well with the experimental data. The tweezers showed angular displacement from 0° to 10° linearly to the deflection of the piezo actuator (0–1.74 mm) with the linearity error of 0.1°. Resonant frequency of the system with/without working tweezer tips was discovered as 101 Hz and 127 Hz, respectively. Our design provides simple and low-cost construction of a versatile manipulator system for samples in the micro/meso-scale (0.1–1 mm).

## 1. Introduction

We herein propose the use of commercially available three-dimensional (3D) printing-based fabrication to create an actuation mechanism that fills the void in sample manipulation between two classes of widely used bioinstruments, nano-scale instruments and macro-scale instruments. Specifically, we targeted manipulation in the size range 100 μm to 1 mm, which we refer to as the micro/meso-scale. Common techniques of nanoscale manipulation include: atomic force microscopy (AFM)-based nanomanipulation systems (target sample range between 200 nm and 10 µm) [1,2,3] and MEMS microgrippers (typical operational range < 100 µm) [4,5,6], which rely on the use of lithography-based micromachined cantilevers or manipulators. On the macro scale, robotic systems such as the Da Vinci Surgical System [7], which targets samples at least 1 mm or larger [8], are built through conventional precision machining [9,10]. In this paper, we demonstrate that the technology of 3D printing is a suitable method to fabricate the mechanical manipulator that occupies the promising area that lies between micromachining and precision machining.

There have been studies of micromanipulators targeting micro/meso-scale samples. Arai et al. demonstrated the capability of a finger-like piezo-electric actuated hybrid micromanipulator for 3D manipulation of microscopic objects by picking up and moving a micro-ball of <10 μm [11]. This design featured baseplates and a number of joints with two chopstick-like glass needles at the end, making it complex to assemble and suffering initial issues with tip alignment. Tabachkova et al. demonstrated the value of shape-memory alloys (SMAs) as an actuator for a micromanipulator. They demonstrated that a 5–14 μm thick TiNiCu alloy ribbon along with an elastic alloy could be used as an actuator in a microtweezer design to manipulate graphene sheets [12]. In this experiment, the micromanipulator exhibited an operational range between 0 nm–1200 nm under the influence of an external source of thermal energy [12]. The major disadvantage of SMAs is the difficulty in controlling displacement because of thermomechanical nonlinearity [13]. Reuben et al. demonstrated the capability of micromanipulation using a pneumatic actuator by manipulating 200 μm zirconium micro-beads. The team proved the potential of pneumatic actuation to produce some of the highest force and power densities of actuation options at the microscale, including a maximum tip opening amplitude of 1 mm and a maximum force output of 50 mN [14]. Pneumatic actuation requires integration of pumps, valves, and tubing, which tend to make the system larger. Here, we report design, fabrication, and testing of a 3D printed, flexure-based actuation mechanism using a piezo bimorph actuator. The mechanism is intended for microtweezers targeted for micro/meso-scale (100 μm to 1 mm) samples. The body of our design is a single 3D printed piece of nylon. The use of 3D printing for flexure-based actuation is a new concept we proposed in our recent study, which not only reduces material costs and scrap by omitting extensive machining but allows for the design of application-oriented, versatile actuation mechanisms. We proved that the flexure works for a nanopositioning stage previously in [15] using 3D printing of Titanium. In a related study, Wei et al. reported on fabrication and testing of a flexure parallel mechanism made of stainless steel 316L [16]. Here, we used polymer to ensure that the piece would be cheap and disposable, which is important for biomedical applications. The use of a piezo bimorph actuator is advantageous because it generates a relatively large deflection (~sub millimeter range), which fits our target range. The 3D printing allows for the easy production of a part that matches specific design requirements. In this case, 3D printing makes it possible to make the cam with a very specific, calculated curve that linearly transforms the deflection of the actuator to the angular displacement of the flexure. The necessary level of precision of cam specifications would be costly with alternative fabrication methods such as milling or drilling.

## 2. Materials and Methods

### 2.1. The Material for 3D Printing

We used 3D printed nylon (PA2200, EOS GmbH, Münich, Germany) provided by Shapeways (New York, NY, USA). We evaluated the characteristics of the materials through fundamental cantilever testing. The specified parameters including Young’s modulus (*E*) and density (*ρ*) could be used to further model the actuation mechanism, specifically the flexure that supports the moving arm of the microtweezers. 

**Figure 1:**
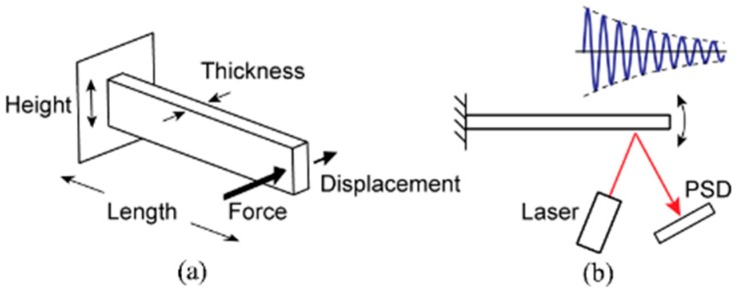
Three-dimensional (3D) printed nylon cantilever diagrams. (**a**) The defined dimensional variables. (**b**) System diagram of the experimental setup used for cantilever resonant frequency analysis.

To test the material properties of the nylon, 3 pieces of 6 different designs (designs #1–#6) were printed, totaling to 18 cantilevers. We used design lengths of 30 mm (#1,2,3) and 40 mm (#4,5,6), and design thicknesses of 0.8 mm (#1 and #4), 1.0 mm (#2 and #5), and 1.2 mm (#3 and #6). The design height was 5 mm for all the cantilevers. Using these cantilevers, we determined the spring constant (*k*) and the resonant frequency (*f*) of each piece. The Young’s modulus (*E*) and the density (*ρ*) of the material were then calculated and compared to the values provided by the material data sheet. Prior to experimental testing, each cantilever was measured for dimensions of length (*l*), height (*h*), and thickness (*t_1_*) using a digitally calibrated caliper and a micrometer (Mitutoyo, Kawasaki, Japan); these dimensions are pictured in Figure 1a. The dimensions of width and thickness were measured in three locations: close point, midpoint, and farpoint. These three measurements were then averaged. Table 1 summarizes the measured values.

The force–displacement relationship was measured in the following way. We applied the force via a simple stepper motor and force-sensing arm connected to a force sensor in a form of a load cell. The force-sensing arm made contact with the cantilever, while movement of the arm was controlled by a serial communication between the stepper motor and MATLAB^®^ through a USB port. The load cell was calibrated by applying 150 incremental steps of 1.60 × 10^−4^ N of known masses. From this, it was determined that the sensitivity of the probe was 0.786 V/µN. Movement of each nylon cantilever was recorded via 30 snapshots from a microscope for 30 corresponding steps of the stepper motor. The recorded 30 images were processed in MATLAB^®^ using a custom script that tracks specified search areas on a set of TIFF files. The cantilever force and the displacement values were then plotted against each other, and a least squares regression line was used to determine the force–displacement slope, which represented the spring constant (*k_cl_*) of the nylon cantilever, which is expressed as:

(1) $$k_{\mathrm{cl}}=\frac{Eht_{1}^{3}}{4l^{3}}$$

Using Equation (1), the Young’s moduli (*E*) could be found using the spring constant (*k_cl_*) in conjunction with the dimensions of length (*l*), height (*h*), and thickness (*t*_1_). The resulting Young’s moduli *E*_1_ from these measurements were more compliant than the expected Young’s modulus of nylon P2200 (1700 MPa) by about a factor of two (see Table 2). This phenomenon was due to the effects of surface porosity of the 3D printed nylon cantilevers. The design thicknesses of the cantilevers (0.8 mm–1.2 mm) were close to the minimum wall thickness (0.7 mm) allowed by the company (Shapeways, New York, NY, USA). As the dimensions of a printed part become smaller, the mechanical characteristics are more affected by the surface porosity. This means that the outermost dimensions of the cantilevers, as measured with a digital micrometer, do not accurately represent the bulk material dimensions and thus the bulk material properties, as we discussed for 3D printed titanium cantilevers in [15]. Characteristics of thin-walled porous 3D printed materials have been reported in literature [17,18]. Differences larger than 20% were reported in [17], and large experimental variations were also observed in [18]. In order to account for the effects of surface porosity, an adjustment of 2*δ* was applied to the thickness dimension of the cantilevers. By applying different values of *δ* to consider the inner thickness and by replacing *t*_1_ in Equation (1) with *t*_2_ *= t*_1_ − 2*δ*, we found that the Young’s moduli *E*_2_ became closest to the datasheet value of 1700 MPa when 2*δ* = 0.23 mm (see Table 2). This indicated the presence of an additional 115 µm of non-contributional porous nylon polymer on each side of the cantilever. The maximum percentage difference between the modified Young’s moduli and the expected Young’s modulus was ~15%, calculated from cantilever design #5.

The dynamic parameters of the nylon material were also measured. We employed a laser that cast a beam onto a reflective piece of aluminum adhered to a secured cantilever; this setup can be seen in Figure 1b. The reflected beam from the aluminum was received by a photodiode position detector (Hamamatsu S5990). When the cantilever was bent, the laser reflection was altered, and thus the reflection on the photodiode was altered, resulting in a change in current. A thin piece of aluminum foil was mounted onto each of the 18 cantilevers using a sticky wax material. The change in weight due to the aluminum strip and the wax material was measured for each cantilever and considered as an additional mass $\Delta m_{\mathrm{cl}}$ to the effective mass when considering the resonant frequency:

(2) $$f_{\mathrm{cl}}=\frac{1}{2\pi }\sqrt{\frac{k_{\mathrm{cl}}}{m_{\mathrm{cl}}+\Delta m_{\mathrm{cl}}}}$$

Here, $m_{\mathrm{cl}}$ is the effective mass of the cantilever approximated as follows:

(3) $$m_{\mathrm{cl}}=\frac{33}{140}\cdot \rho t_{2}lh$$

Note that $\rho t_{2}lh$ in Equation (3) is the actual mass of the cantilever and requires the cantilever dimensional measurements to be found. To be consistent, the inner thickness (*t*_2_) was used to consider the bulk volume in Equation (3). One-by-one, the 18 cantilevers were secured onto this experimental setup and given an impulse via a thin metal rod flicked on the free-moving end of the cantilever. The signals from the position detector were recorded with a National Instruments myDAQ device at a sampling frequency of 10 kHz for 2 s. The recorded signal of each cantilever was analyzed in MATLAB^®^ using an envelope mapping function with an exponential curve-fitting algorithm to determine the decay time constant (*τ*), and a single sided amplitude spectrum Fast Fourier transform (FFT) to determine the signal’s resonant frequency ($f_{\mathrm{cl}}$). The equations used for exponential curve fitting and the calculation of the decay time constant (*τ*) can be seen in Equations (4) and (5), respectively.

(4) $$y\left(t\right)=a\cdot e^{-bt}+c$$

(5) $$\tau =\;\frac{1}{b}$$

In order to find the density (*ρ*) of the 3D printed nylon material, Equations (2) and (3) were deployed. The resulting densities (*ρ* = 0.84 ± 0.24 g/cm^3^) were comparable to the PA 2200 material data sheet’s value of 0.93 g/cm^3^ (see Table 3). These experimentally calculated densities were also compared against the average of three 3D printed cubes each sized at 1 cm^3^ with an average density of 0.953 g.

### 2.2. Piezo Basic Characterization

**Figure 2:**
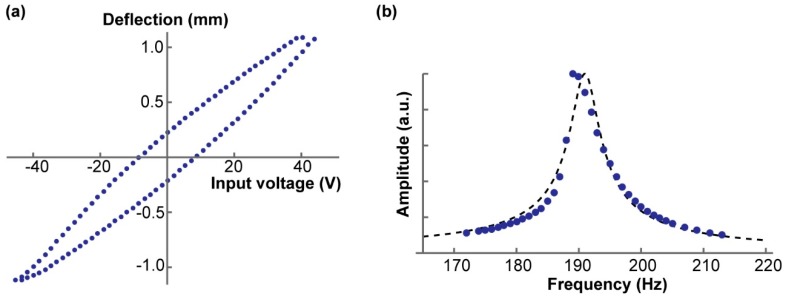
Characteristics of the piezoelectric bimorph actuator. (**a**) The applied voltage to the piezoplate versus the piezoplate deflection. Typical non-linear characteristic of piezoelectric material was quantified. (**b**) The frequency response of the piezo actuator given an initial input frequency and measuring the corresponding output amplitude. The dotted line is the curve fit to the theoretical frequency response of a damped harmonic oscillator. This graph allowed for the calculation of resonant frequency ($f_{\mathrm{pz}}$), effective mass ($m_{\mathrm{pz}}$), Q factor ($Q_{\mathrm{pz}}$), and damping coefficient ($c_{\mathrm{pz}}$).

This system requires piezo actuation as input and transforms this motion into tweezer angular displacement for sample handling. We used a piezoelectric bimorph actuator sized 40 mm × 10 mm × 0.5 mm (Steminc, Doral, FL, USA). The piezoplate was controlled via the output voltage of an 8-bit digital-to-analog converter (DAC) whose voltage ranged between 0–5 V. This output voltage was then amplified in the range of −45 V to 45 V using a high voltage operational amplifier (Analog Devices, ADA4700, Norwood, MA, USA). A characterization of the relationship between the applied voltage to the piezoplate and the corresponding deflection of the piezoplate can be seen in Figure 2a. 

As with the 3D printed nylon cantilevers, the same method of analysis was employed to find the spring constant of the piezoplate. After incremental application force and optical analytics, the spring constant (*k*_pz_) was determined to be 565 N/m. The frequency response was measured by applying a sinusoidal voltage input to the piezoplate ranging in frequency from 1 Hz to 310 Hz. A small piece of aluminum foil was mounted onto the piezoplate, and the change in mass ($\Delta m_{\mathrm{pz}}$) was measured.

The resonant frequency ($f_{\mathrm{pz}}$) of the piezoplate was measured to be 189 Hz. With the resonant frequency and the spring constant of the actuator, Equation (6) was used to find the effective mass of the piezoplate ($m_{\mathrm{pz}}$).

(6) $$f_{\mathrm{pz}}=\;\frac{1}{2\pi }\sqrt{\frac{k_{\mathrm{pz}}}{m_{\mathrm{pz}}+\;\Delta m_{\mathrm{pz}}}}$$

The Q factor ($Q_{\mathrm{pz}}$) of the frequency response could then be calculated generally by locating the position where the output voltage dropped to 1/√2 of the maximum output voltage. Through the above listed methods, the Q factor of the piezo plate was calculated to be 47. Finally, using Equation (7), the damping coefficient (i.e., the viscous damping coefficient) was found to be 0.010.

(7) $$c_{\mathrm{pz}}=\frac{\sqrt{\left(m_{\mathrm{pz}}+\;\Delta m_{\mathrm{pz}}\right)\cdot k_{\mathrm{pz}}}}{Q_{\mathrm{pz}}}$$

### 2.3. Design of the Actuation Mechanism

**Figure 3:**
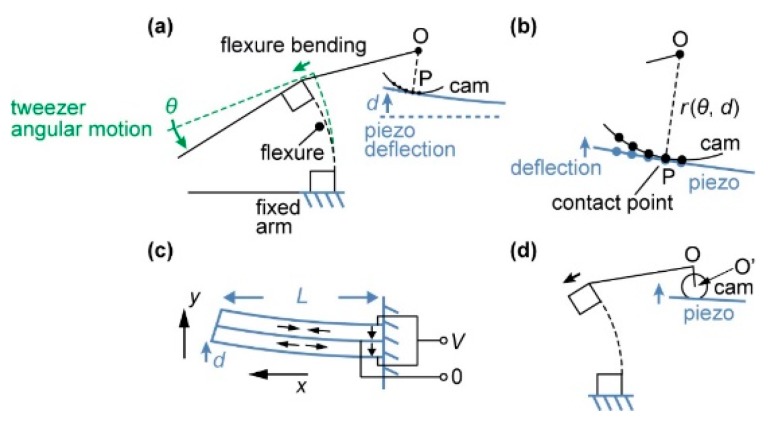
Principle of the displacement transmission mechanism. The cam is designed for *θ* to be linearly correlated to *d*. (**a**) The moving arm of the tweezers is supported by a flexure and actuated by a piezo bimorph actuator. (**b**) The curve of the cam is given as a path traced by the point of contact. (**c**) Piezo bimorph actuator works in a similar way to a bimetal cantilever (**d**) The cam is approximated as a circle in the actual 3D design (see Supplementary Video S1).

Figure 3 shows the principle of the displacement transmission mechanism we developed for the tweezers. The moving arm of the tweezers is supported by a flexure and moved by a piezo bimorph actuator. A cam is designed to linearly transfer the deflection (*d*) of the actuator to the angular displacement (*θ*) of the flexure. As shown in Figure 3a,b, the curve of the cam is given as the path, which the tangent point P traces as *d* and *θ* increase. In this theoretical model, the contact point moves by pure rolling without sliding, minimizing the friction between the cam and the actuator. For each combination of *d* and *θ*, the cam-piezo contacting point, P, is given as the closest point on the piezo to the point O, which is a fixed point within the cam (Figure 3b). The vector $\vec{\mathrm{OP}}\;$ is given as a function of *d* and *θ*:

(8) $$\vec{\mathrm{OP}}\;=\;r\left(\theta ,\;d\right)\;$$

The plot of Equation (8) provides the trace of the contact point, which equals the curve of the cam. Here, we assumed the piezo-bimorph actuator to be a simple bi-layer cantilever, where one-layer contracted and the other expanded, resulting in a bending motion on application of a voltage (Figure 3c). With this assumption, the bending of the piezo actuator is expressed as:

(9) $$y\;=\;d\;{\left(\frac{x}{L}\right)}^{2}$$

Because of the hysteresis, application of the same voltage did not result in the same deflection of the piezo actuator, as shown Figure 2a. It was practical to linearly correlate the angular displacement with the piezo mechanical deflection rather than the piezo control voltage. It was desired that the flexure bending angle *θ* should be linearly correlated with the piezo deflection *d*, to be expressed as follows:

(10) θ = k·d

When Equation (10) was assumed, $\vec{\mathrm{OP}}\;=\;r\left(\theta ,\;d\right)$ was given as a function of *θ* with *k* as a constant. The value of *k* should be so chosen such that magnitude of **r**, i.e., the length of OP, is a monotonically decreasing function of *θ*. We used a custom Mathematica™ program to find the position of P and find $r=\vec{\mathrm{OP}}$ as a function of *θ*. Once the curve **r**(*θ*) was found, curve fitting was used to find a circle closest to the curve. As shown in Figure 3d, the center O*’* of the fitted circle is usually different from O, which is used to find the curve. Calculated motion of this design is shown in the Supplementary Video S1. Note that it is also possible to design a cam that converts the piezo deflection to a linear displacement measured in the y-axis or in the tip-to-tip direction. However, linear displacement is highly dependent on the tip design and how the pair of tips are arranged, and a cam needs to be designed for each specific experimental configuration. We chose the angular displacement as a design parameter, because it is easily applicable to multiple, general experimental conditions.

### 2.4. System Design

**Figure 4:**
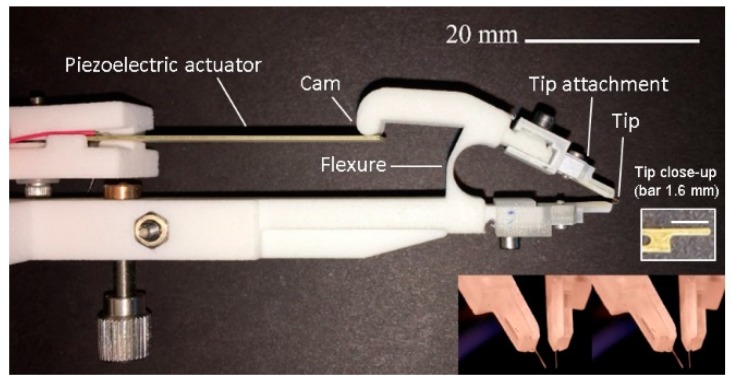
Photograph of the tweezer system.

**Figure 5:**
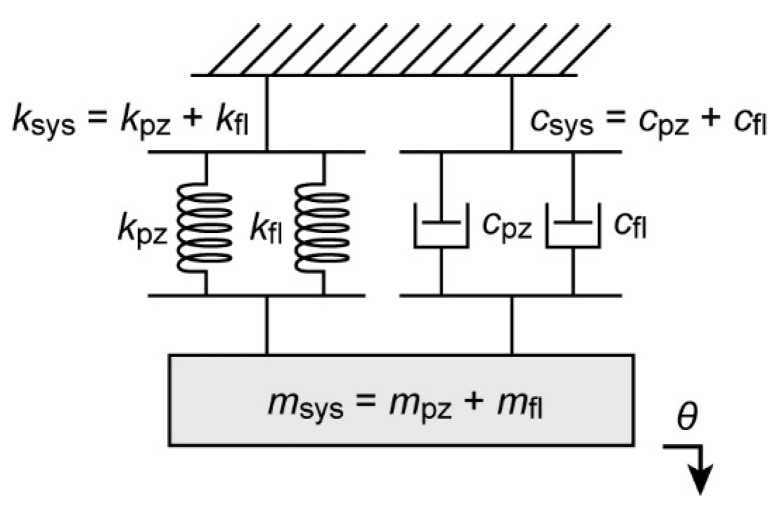
System block diagram.

Figure 4 shows a photograph of the completely constructed system, which includes the flexure, the piezoelectric bimorph actuator, and the working tweezer tips. The width and the length of the flexure are 4 mm and 10 mm, respectively. The designed thickness of the flexure’s thinnest part is 0.8 mm. Additionally, the lower right corner shows a photograph of the microtweezer with tips fully open (corresponding to a −45 V supply to the piezoelectric bimorph actuator). In Figure 5, it can be seen that a simplified system block diagram consists of a linear model spring dashpot system, also commonly referred to as the damped harmonic oscillator [19,20]. In this model, the associated spring constant of the system ($k_{\mathrm{sys}})$ is approximated as the summation of the spring constant of the tweezer flexure ($k_{\mathrm{fl}}$) and the spring constant of the piezo actuator ($k_{\mathrm{pz}}$). The other portion of the damped harmonic oscillator model is a viscous dashpot. Similarly to that of the spring constant, the associated damping coefficient of our system ($c_{\mathrm{sys}}$) is the summation of the damping coefficient of the tweezer flexure ($c_{\mathrm{fl}}$) and the damping coefficient of the piezo actuator ($c_{\mathrm{pz}}$). Just the same as the representation of the piezo-tweezer system effective mass ($m_{\mathrm{sys}}$) is the summation of the effective mass of the tweezer-flexure ($m_{\mathrm{fl}}$) and the effective mass of the piezo actuator ($m_{\mathrm{pz}}$). 

Tweezer tips (L = 1600 µm, W = 150 µm, and t = 25 µm) were patterned from a 25 µm-thick brass film (C260 brass, McMaster-Carr, Elmhurst, IL, USA) through standard lithography. The brass film was first sandwiched between two photoresist films (Micromark, Berkeley Heights, NJ, USA), the topside of which was patterned through UV exposure and development. The tweezer tips were then etched out with a ferric chloride solution (Micromark). A similar process was reported in [21] for the fabrication of AFM cantilever. The attachment for the tips were 3D printed from UV curable acrylic polymer (Shapeways) to hold the tips at the end of the arms. Tweezer tips could be easily replaced for each experiment. A close-up photograph of the brass tip is shown in a panel in Figure 4. The measured average spring constant of 6 probes was 1.3 ± 0.1 N/m.

## 3. Results

### 3.1. Comparison of Computational and Experimental Models 

**Figure 6:**
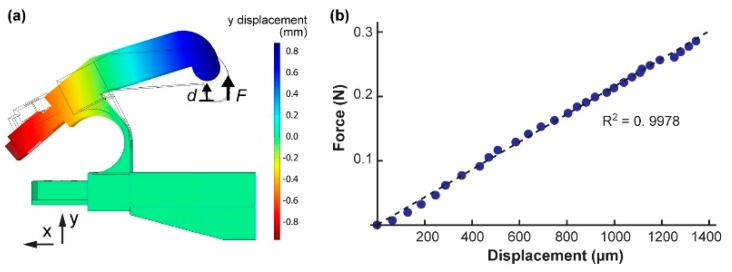
Once fundamental material properties are found, characteristics of an arbitrarily designed part can be found from a finite element method (FEM) analysis. (**a**) COMSOL^®^ analysis of the tweezer flexure. The image displays the cam with an applied force of 0.2 N. (**b**) Experimentally measured tweezer flexure force vs. displacement curve.

We performed a finite element method (FEM) analysis to show that the experimentally discovered parameters could be adequately used to find the spring constant ($k_{\mathrm{fl}}$) and the resonant frequency ($f_{\mathrm{fl}}$) of an arbitrarily designed 3D printed part. The material properties used were those of the 3D printed nylon (PA2200) we showed in the previous section (Young’s Modulus of 1700 MPa and the average density of 0.84 g/cm^3^). The designed width of the tweezer flexure was 0.8 mm. As previously explained, the size of this dimension was reduced during computation by 0.23 mm to more accurately represent the bulk characteristics of the material for COMSOL^®^ analysis (Stockholm, Sweden) (see Figure 6a). The COMSOL^®^ analysis yielded a spring constant equal $k_{\mathrm{fl}\_\mathrm{COMSOL}}$ = 240 N/m. The resonant frequency from the COMSOL^®^ analysis was $f_{\mathrm{fl}\_\mathrm{COMSOL}}$ = 111 Hz. 

Using the same force testing set up as the 18 cantilevers, the spring constant of the tweezer flexure was experimentally found to be $k_{\mathrm{fl}\_\mathrm{measured}}$ = 215 N/m with an applied force range of 0 N to 2.9 N (see Figure 6b). The difference between the experimental value and the COMSOL^®^ simulated value was 25 N/m (percentage difference ~11%). Using a laser, an adhered piece of reflective aluminum, and a photodiode position detector, the resonant frequency of the tweezer flexure was experimentally determined. The resonant frequency of the tweezer flexure was found to be $f_{\mathrm{fl}\_\mathrm{measured}}$ = 121 Hz. These two frequencies had a difference of 10 Hz (percentage difference ~9%). The measured Q factor was ($Q_{\mathrm{fl}}$ = 170). The resonant frequency, the spring constant, the effective mass, and the damping coefficient followed the same relationship as we described for the piezoactuator:

(11) $$f_{\mathrm{fl}}=\;\frac{1}{2\pi }\sqrt{\frac{k_{\mathrm{fl}}}{m_{\mathrm{fl}}+\Delta m_{\mathrm{fl}}}}$$

(12) $$c_{\mathrm{cl}}=\;\frac{\sqrt{\left(m_{\mathrm{fl}}+\Delta m_{\mathrm{fl}}\right)\cdot k_{\mathrm{fl}}}}{Q_{\mathrm{fl}}}$$

Using Equations (11) and (12), we found the tweezer flexure’s effective mass ($m_{\mathrm{fl}}$ = 0.37 g) and the damping coefficient ($c_{\mathrm{fl}}$ = 0.0035). 

### 3.2. System Performance 

**Figure 7:**
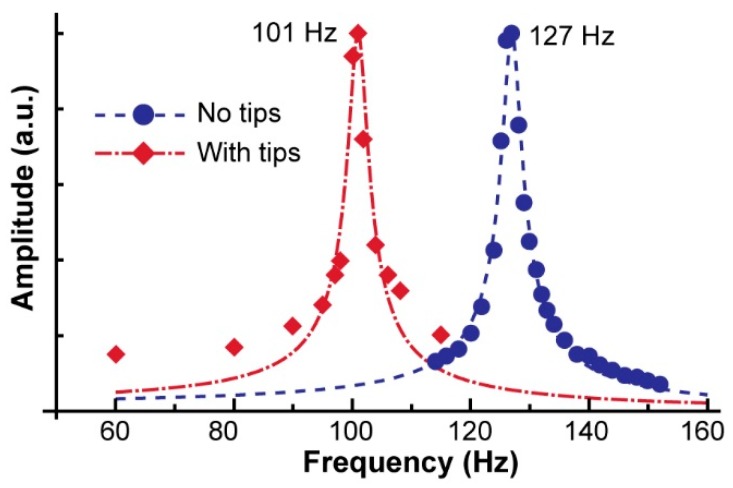
Frequency response of the system. The series in blue shows the frequency response of the piezo-tweezer system. The orange series is for the system with the attached tips. The resonant frequencies of 101 Hz and 127 Hz with and without tips, respectively, are indicated.

The 3D printed tweezer flexure, the piezo actuator, and the total system were characterized to determine the system frequency response. The frequency response was measured by applying a sinusoidal voltage input to the piezoplate ranging in frequency from 1 Hz to 310 Hz. A small piece of aluminum foil was mounted onto the outer surface of the tweezer’s cam. As previously described, a laser was applied to the aluminum mount, which reflected onto a photo-detector. We conducted this experiment for the system frequency response including and excluding the detachable tweezer tips (these tips vary based on project application). The results of this experiment are depicted in Figure 7. Because the spring constant of the working tip (1.3 N/m) was less than 1% of that of the flexure (215 N/m), the resonant frequency should not have showed measurable changes, even when the working tips were touching an object. However, when the tip was immersed in water, we observed a slight effect of damping, and the peak oscillating amplitude was reduced to ~90%, which corresponded to a ~10% increase in the system damping coefficient.

**Figure 8:**
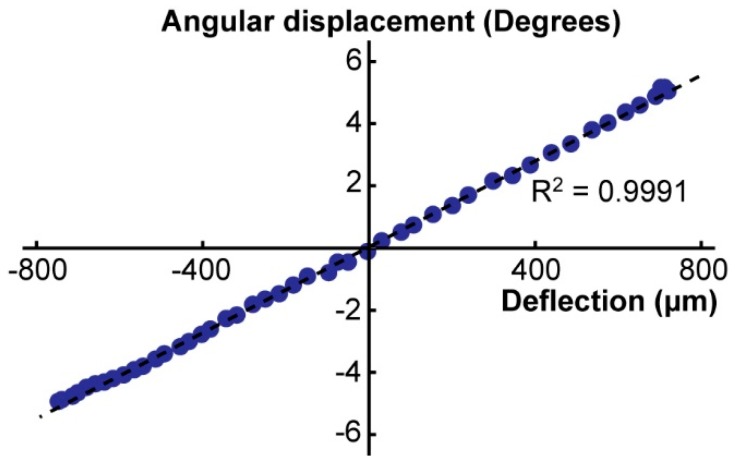
The above graph displays the angular displacement (degrees) of the tweezer tips plotted against the corresponding piezoplate displacement (m).

In addition to the frequency characterization, we demonstrated that the actuation mechanism of the cam and the flexure linearly transferred the piezo deflection to the angular displacement of the tweezer tips within the full operational range of the actuator. The result is shown in Figure 8. The root-mean-square error from the linear fit was 0.1°.

If we consider the simplified model as illustrated in Figure 5, the spring constant, the effective mass, and the damping coefficient of the system can be approximated as the summations of those of the tweezer flexure and the piezo actuator. When we use the values obtained in the previous sections, we find:

(13) $$k_{\mathrm{sys}}=k_{\mathrm{fl}}+k_{\mathrm{pz}}=215+565=780\;N/m$$

(14) $$m_{\mathrm{sys}}=m_{\mathrm{fl}}+m_{\mathrm{pz}}=0.37+0.40=0.77\;g$$

(15) $$c_{\mathrm{sys}}=c_{\mathrm{fl}}+c_{\mathrm{pz}}=0.0035+0.010=0.014\;\mathrm{Ns}/m$$

When we use these values, the expected resonant frequency ($f_{\mathrm{sys}}$) is calculated as:

(16) $$f_{\mathrm{sys}}=\frac{1}{2\pi }\sqrt{\frac{k_{\mathrm{sys}}}{m_{\mathrm{sys}}}}=160\;\mathrm{Hz}$$

which is comparable to the measured value of $f_{\mathrm{sys}}=127\;\mathrm{Hz}$. When we use (13), (14), and the measured Q factor for the system without tips ($Q_{\mathrm{sys}}$ = 38),

(17) $$c_{\mathrm{sys}}=\;\frac{\sqrt{m_{\mathrm{sys}}\cdot k_{\mathrm{sys}}}}{Q_{\mathrm{sys}}}=0.020\;\mathrm{Ns}/m$$

which is also comparable to the expected value shown in Equation (15). 

## 4. Conclusions

We showed the design of an actuation mechanism based on a 3D printed flexure and a piezo bimorph actuator. The elastic modulus and the density of the 3D printed nylon were first determined by studying sets of simple cantilevers. The finite element method (FEM) analysis of the tweezers on COMSOL™ based on the obtained fundamental data matched well with the measurement. The angular displacement of the moving arm showed a good linearity (R^2^ = 0.9991) with the linearity error of 0.1 degrees within the actuator deflection (0–1.74 mm), showing the efficacy of the 3D printed cam. We already utilized this manipulation systems in many successful applications we reported elsewhere [22,23,24]. In these previous studies, we used the tweezers for relative positioning with incremental tip displacement. The smallest positioning step was defined by the resolution of the voltage step sent from the controller, and the typical minimum operational step was ~1.7 μm/step at a voltage application of 0.35 V/step. In this paper, we described the detailed design principle and the material and the dynamic characteristics of the manipulation system. Our microtweezer design is a low-cost, efficient method of the direct assembly method of micro/meso-scale biosamples.

## Figures and Tables

**Table 1:** Dimensional Measurements: 3D Printed Nylon Cantilevers.

Design	*l* (mm)	*h* (mm)	*t*_1_ (mm)	*t*_2_ (mm)
#1	30.2	5.11	0.806	0.576
#2	29.9	5.11	0.879	0.649
#3	29.8	5.12	0.944	0.714
#4	40.0	5.14	1.00	0.774
#5	40.2	5.14	1.07	0.840
#6	40.3	5.12	1.13	0.901

**Table 2:** Static Analysis: 3D Printed Nylon Cantilevers.

Design	*k* (N/m)	*E*_1_ (MPa)	*E*_2_ (MPa)	Expected *E* (MPa)
#1	15.7	623	1,700	1,700
#2	20.6	637	1,600	1,700
#3	30.4	714	1,650	1,700
#4	39.0	796	1,730	1,700
#5	55.9	961	1,990	1,700
#6	65.6	961	1,910	1,700

**Table 3:** Resonant Frequency Analysis: 3D Printed Nylon Cantilevers.

Design	*f* (Hz)	*τ*	*ρ* (g/cm^3^)
#1	143	2047.8	0.584
#2	193	1909.4	0.586
#3	239	1447.2	1.25
#4	77	4384.2	0.866
#5	90.9	2727.3	0.875
#6	127	2335.2	0.866

## Supplementary Materials

The following are available online at https://www.mdpi.com/2072-666X/10/7/470/s1. Video S1: Cam design and actuation.avi.
